# Standard protocol devised by the Japanese Pediatric Impedance Working Group for combined multichannel intraluminal impedance-pH measurements in children

**DOI:** 10.1007/s00595-019-01833-6

**Published:** 2019-06-18

**Authors:** Suguru Fukahori, Hisayoshi Kawahara, Takanori Oyama, Takeshi Saito, Ryuichi Shimono, Aya Tanaka, Takuo Noda, Reiko Hatori, Junko Fujino, Minoru Yagi, Japanese Pediatric Impedance Working Group (Japanese-PIG)

**Affiliations:** 1 Department of Pediatric Surgery, Kurume University School of Medicine, 67 Asahi-machi, Kurume, Fukuoka 830-0011 Japan; 2 Department of Pediatric Surgery, Hamamatsu University School of Medicine, Shizuoka, Japan; 3 Department of Pediatric Surgery, Okayama University, Okayama, Japan; 4 Department of Pediatric Surgery, Chiba University Graduate School of Medicine, Chiba, Japan; 5 Department of Pediatric Surgery, Kagawa University, Kagawa, Japan; 6 Department of Pediatrics, Gunma University Graduate School of Medicine, Gunma, Japan; 7 Department of Pediatric Surgery, Saitama Medical Center, Dokkyo Medical University, Saitama, Japan

**Keywords:** Multichannel intraluminal impedance-pH measurements, Standard protocol, Gastroesophageal reflux, Children

## Abstract

Multichannel intraluminal impedance-pH measurements (MII-pH) are useful for evaluating acid and non-acid gastroesophageal reflux (GER). However, the use of MIH-pH is not yet established in Japan. The Japanese Pediatric Impedance Working Group (Japanese-PIG) convened to devise a standard protocol for MII-pH in Japanese children. The expert members of the Japanese-PIG collected data on pediatric MII-pH from the relevant literature in English, including the standard protocol of MII-pH presented by the European PIG, and the insights of international experts. The resultant consensus was included in the contents of the standard protocol of MII-pH. The standard protocol included standardization of the indication, methodology, and interpretation of MII-pH in Japanese children. The criteria for abnormal GER by MII-pH were defined using the Reflux Index and number of total reflux episodes independently in children aged < 1 year and those aged ≥ 1 year. Moreover, a significant relationship between GER and symptoms was identified using the symptom index and symptom association probability approach. We conclude that the current version of the protocol for MII-pH is tentative because it is not based on data from Japanese children. Further studies are needed to render this protocol clinically beneficial and expand its use in Japan.

## Introduction

Esophageal pH monitoring has been the gold-standard method for the diagnosis of gastroesophageal reflux (GER) disease in children. However, multichannel intraluminal impedance-pH measurements (MII-pH), which can be used to assess both acid and non-acid GER, have been implemented in clinical practice in Western countries. In 2014, the 24-h MII-pH technique was granted pharmaceutical affairs permission in Japan and it is now being applied in clinical practice. In 2012, the European Pediatric Impedance Working Group (Euro-PIG) of the European Society for Pediatric Gastroenterology, Hepatology, and Nutrition presented a standard protocol for the measurement of MII-pH [1]. However, the methodology and analysis approach for MII-pH have not been established in Japan. Therefore, we need to produce the Japanese version of the standard protocol for MII-pH.

In 2015, the Japanese Pediatric Impedance Working Group (Japanese-PIG) was established by the Japanese Pediatric Gastrointestinal Motility Society, aiming to produce the Japanese version of the standard protocol for MII-pH.

## Methods

The expert members of the Japanese-PIG collected data on pediatric MII-pH from the relevant literature published in English, including the standard protocol of MII-pH presented by the Euro-PIG, and the insights of international experts. Following extensive discussion among the members, the Japanese-PIG produced a protocol for the standardization of the indications, methodology, and interpretation of MII-pH in Japanese children. The criteria for abnormal GER were defined according to the data presented in recent literature, involving international pediatric patients suspected of having pathological GER.

## Results

### Utility of and indications for MII-pH

MII-pH can detect the movement of liquid, solid, and gas contents through changes in intra-esophageal impedance. It can also evaluate acid and non-acid reflux from the stomach to the esophagus and be performed under medication with acid secretion inhibitors. Moreover, it can detect non-acid GER under conditions of high gastric pH. MII-pH is indicated for examination of the causal relationship between symptoms and GER in children with esophageal symptoms such as nausea, vomiting, heartburn, or abdominal pain; or extra-esophageal respiratory symptoms such as cough, wheeze, decreased oxygen saturation, frequent respiratory tract infections, otitis media, or apneic spells; or neurological symptoms such as oral feeding difficulty or exacerbation of seizures; and other symptoms such as failure to thrive or anemia. It may be used to evaluate symptoms caused by GER, or to evaluate GER in children whose symptoms do not improve after anti-reflux surgery. The MII-pH technique is especially useful for evaluating the relationship between intermittent symptoms, such as cough, stroke, and non-acid GER.

### Examination

#### Device

**Fig. 1:**
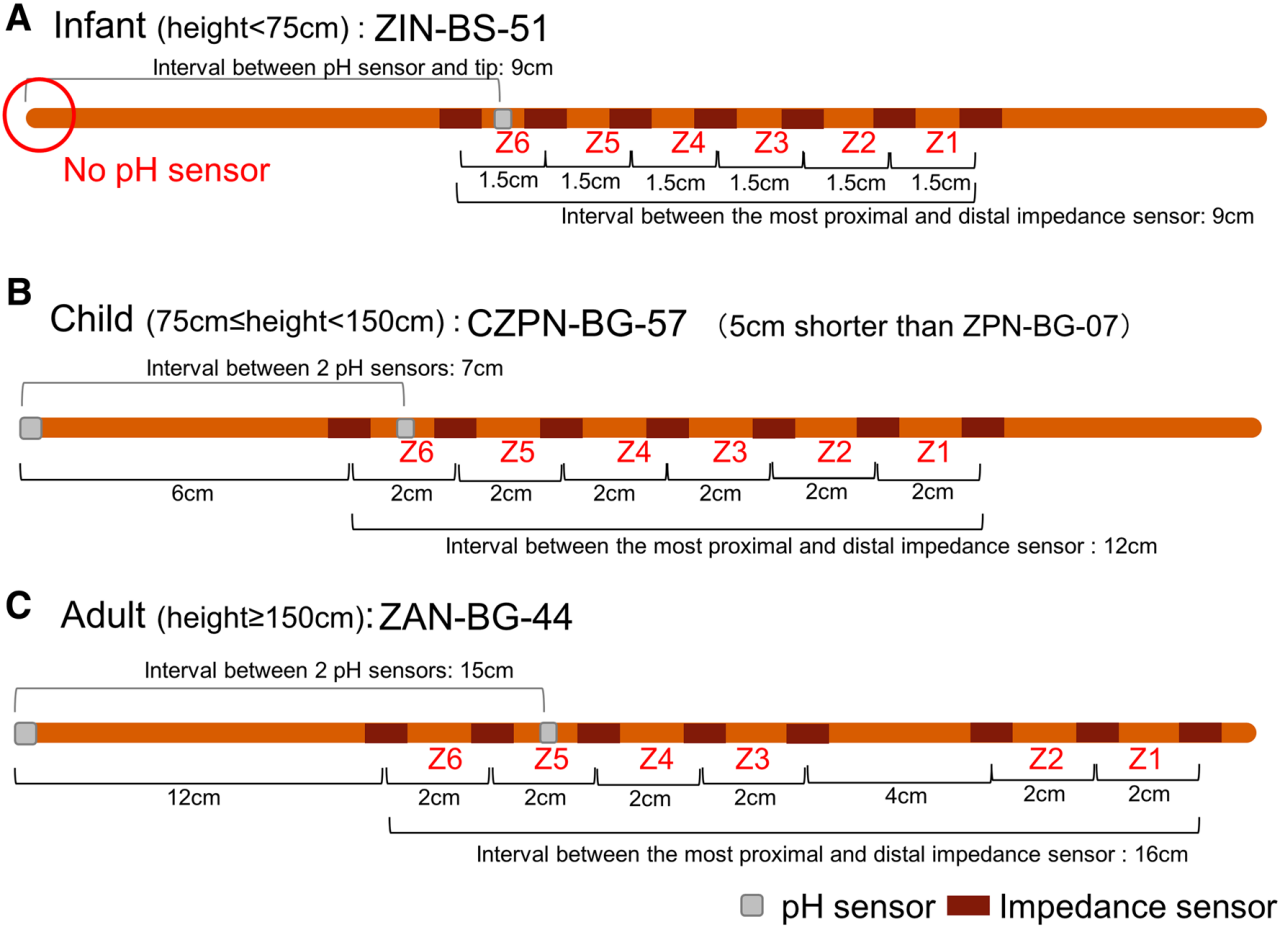
Types of catheters used for MII-pH measurements in pediatric and adult patients. **a** Infant catheter (ZIN-BS-51): the impedance channels are distributed at 1.5-cm intervals and the pH sensor is positioned at the center of the most distal interval of the impedance channels. **b**, **c** Pediatric and adult catheters: the impedance channels are distributed at 2-cm intervals, and the pH sensor is positioned at the center of the most distal (CZPN-BG-57, ZPN-BG-07) or the second most distal interval of the impedance channels (ZAN-BG-44). The layouts of the sensors on CZPN-BG-57 and ZPN-BG-07 are identical, except for the position of the pH sensor. The distance between the 2 pH sensors in CZPN-BG-57 and ZPN-BG-07 is 7 cm and 12 cm, respectively

Presently, the Sleuth ZepHr™ (Sandhill Scientific, Inc., Highlands Ranch, CO, USA) is the only commercially available device for the measurement of MII-pH, approved in 2014 under the pharmaceutical affairs law in Japan. A disposable catheter of appropriate length with six impedance channels and one or two antimony pH sensors should be used for each examination. The following catheters of appropriate length are commercially available in Japan from Sandhill Scientific, Inc. (Fig. 1):

- Infant (height: < 75 cm): ZIN-BS-51 (1-ch pH)
- Child (height: ≥ 75 to < 150 cm): CZPN-BG-57, ZPN-BG-07 (2-ch pH)
- Adult (height: ≥ 150 cm): ZAN-BG-44 (2-ch pH).

The distance between the impedance channels and the position of the pH sensor differ among the catheters as follows:

- Infant catheter (ZIN-BS-51): the impedance channels are distributed at 1.5-cm intervals and the pH sensor is positioned at the center of the most distal interval of the impedance channels.
- Pediatric and adult catheters: the impedance channels are distributed at 2-cm intervals and the pH sensor is positioned at the center of the most distal (CZPN-BG-57, ZPN-BG-07) or second most distal interval of the impedance channels (ZAN-BG-44).

The layouts of the sensors on the CZPN-BG-57 and ZPN-BG-07 are identical, except for the position of the pH sensor. The distance between the two pH sensors in the CZPN-BG-57 and ZPN-BG-07 is 7 cm and 12 cm, respectively. These two catheters should be used based on the placement position of the tip of the pH sensor. When ZPN-BG-07 is used for a small child with a tiny stomach, the tip of the catheter possibly touches the wall of stomach, which can displace the intra-esophageal pH sensor. Therefore, the CZPN-BG-57 is preferable for an infant or toddler.

#### Instructions prior to the measurement of MII-pH

Informed consent after disclosure of potential risks such as complications caused by failure of the catheter or device, catheter tip misplacement, and mucosal damage, should be provided by the parents or guardian of the child prior to measurement of the MII-pH. Medications that could affect measurement results, such as H_2_ blockers, proton pump inhibitors, and prokinetic agents, should be discontinued at least 3 days prior to the examination. If discontinuation of these medications is not possible for medical reasons; this should be stated in the analysis report. However, this recommendation shall not apply when the examination is performed to evaluate the effect of these medications. To avoid vomiting or aspiration, the child should be fasted for ≥ 3 h prior to insertion of the catheter.

#### Preparation of the examination

The pH electrode of the MII-pH catheter should be calibrated according to the instructions provided by the manufacturer. Catheters produced by Sandhill Scientific, Inc., should be calibrated to between pH 4 and 7 after soaking in pH 4 solution or tap water for ≥ 10 min. Catheters for which the pH electrode and the impedance sensors show unstable values during the calibration process should not be used due to the risk of yielding false abnormal MII-pH values.

#### Insertion and placement of the MII-pH catheter

Sedatives that may affect the results of the examination should be avoided; however, a local anesthetic agent such as intra-nasal anesthetic spray may be used for the trans-nasal insertion of the MII-pH catheter. If the catheter is inserted under general anesthesia, the examination should be initiated after the patient has completely recovered to a normal physical condition. Lidocaine gel is often used as a lubricant during catheter insertion. Of note, according to the instructions provided by Sandhill Scientific, Inc., this agent may disturb the accuracy of the antimony pH sensor. The pH sensor on the MII-pH catheter should be placed in the esophagus, two vertebrae above the base of the diaphragm. It is recommended that the placement of the pH sensor is confirmed under fluoroscopic guidance. If fluoroscopic guidance is unavailable [2], the distance from the external naris to the lower esophageal sphincter should be calculated using the Strobel formula (0.252 × body length [cm] + 5) to check the placement of the pH sensor. The drawback of this formula is that the greater the height of the patient, the greater the overestimation of the length of the esophagus, because it is the infant version.

When the placement of the MII-pH catheter has been checked using the Strobel formula, the positions of the pH and impedance sensors should be checked radiographically. Moreover, prior to removal of the MII-pH catheter, it is necessary to confirm the position of the pH and the impedance sensors fluoroscopically, as they can occasionally move or coil up during the examination.

#### Recording during the examination

Prior to the examination, the physician should instruct the patient, caregiver, or nurse to push the three buttons in front of the MII-pH machine appropriately, when a GER-related symptom is observed during the examination. It is imperative that they recognize the importance of pushing these buttons without delay to accurately evaluate the relationship between the targeted symptom and GER. To ensure the accuracy of the MII-pH examination, in addition to assessing the duration of meals from start to finish, the body position (upright/supine), and wake–sleep cycle, the physician should instruct the patient, caregiver, or nurse to record the symptoms and medical care given during the examination in a diary.

#### Points to note during the examination

The recommended duration of the MII-pH examination for diagnostic purposes is 24 h, to account for differences in the incidence of GER between the postprandial and fasting periods, the upright and supine positions, and the wake–sleep cycle. The consumption of carbonated beverages should be avoided because the gas may complicate the data analysis. Moreover, as hot or cold beverages or foods may affect the incidence of GER, the patient’s meals during the examination should comprise the same contents as usual.

### Analysis

#### Transfer of the recorded MII-pH data to a computer

After the examination, the MII-pH data in the recording medium are downloaded into a personal computer and analyzed using a specialized software program (BioView™; Sandhill Scientific, Inc.).

#### Addition of the diary information

The data recorded in the diary, such as symptoms and medical care, are added to the downloaded data. If a symptom button was pushed during the examination, the timing should be checked against the diary record. If an abnormal waveform is noted in the recorded MII-pH data, the duration, including the abnormal data, should be excluded from the analysis. Of note, only abnormal MII-pH data can be selectively excluded.

#### Term

- Reflux episode (RE)
  - Liquid RE
    Liquid RE is defined as an event in which the impedance values of at least two consecutive distal sensors decrease by > 50% of the average 3-s mean baseline value prior to the initiation of RE and continue for > 2 s in the most distal channel. The duration of the RE is the time until the impedance values return to ≥ 50% of the 3-s mean baseline value.
  - Acid RE
    Acid RE is bolus movement observed using the impedance sensors as reflux to the esophagus in a retrograde direction in two consecutive channels of the bottom three channels and a nadir intra-esophageal pH < 4.0.
- Non-acid RE
  Non-acid RE is bolus movement observed using the impedance sensors as reflux to the esophagus in a retrograde direction in two consecutive channels of the bottom three channels, with a pH ≥ 4.0.
- Gas RE
  Gas RE is defined as an event in which the impedance values of two consecutive sensors increase sharply by > 50% of the mean baseline value prior to initiation of the RE, with at least one showing > 5000 Ω.
- Mixed RE
  Mixed RE is defined as an event in which the liquid and gas RE occur simultaneously.
- Proximal (extent) RE
  Proximal (extent) RE is defined as an event that reaches the most proximal one or two sensors.
- pH-only RE
  pH-only RE is defined as pH RE in the absence of retrograde bolus movement.

- Exposure time
  - Acid exposure time
    Total time of acid clearance during the measurement period.
  - Percent time (Reflux Index)
    The percentage of acid exposure time during the esophageal pH measurement period.
  - Bolus exposure time
    Total time of bolus clearance during the measurement period.
- Bolus exposure index
  The percentage of bolus exposure time during the MII-pH measurement period.

- Clearance time
  - Acid (chemical) clearance time
    The amount of time during which the pH is < 4.0. The BioView™ software program automatically calculates the average acid clearance time.
  - Bolus clearance time
    Elapsed time during which the bolus is present at each channel level in the course of a RE. Measured by MII as the elapsed time between bolus entry and bolus clearance. The BioView™ software program automatically calculates the median bolus clearance time.

- Indicators showing a relationship between symptoms and GER
  - Symptom index (SI) = number of GER-related symptoms/total number of symptoms
    GER-related symptoms are those experienced within 5 min after the RE. A SI > 50% indicates a significant relationship between the symptom and GER [3]. However, the SI tends to yield a false-positive result if the total number of symptoms is small or the number of GER-related symptoms is large. The SI is characterized by high sensitivity and low specificity.
  - Symptom sensitivity index (SSI) [4] = number of symptom-related GER episodes/total number of GER episodes
    A symptom-related GER episode is defined as GER that occurs within 5 min prior to the symptom. A SSI > 10% indicates a significant relationship between the symptoms and GER [5]. However, the SSI tends to show a false-positive result if the number of GER-related symptoms is high or the number of GER episodes is low.
  - Symptom association probability (SAP) = (1.0 − *p* value) × 100%
    The total measurement time is divided into 2-min intervals (time windows), and all time windows are categorized into four groups: GER (+) and symptom (+), GER (+) and symptom (−), GER (−) and symptom (+), GER (−) and symptom (−). The *p* value is calculated by Fisher’s exact probability test, using the aforementioned formula. A SAP > 95% indicates a significant relationship between the symptom and GER [6]. The numbers of GER episodes and symptoms do not affect the outcome of SAP greatly. Thus, the SAP is recognized as the most robust statistical approach to analyze the relationship between symptoms and GER. However, the lowest required number of symptoms to yield reliable SAP results has not been defined and this number is considered to vary depending on the type of symptoms.

#### Auto-analyses (AutoScan™)

When using the AutoScan™ (Sandhill Scientific, Inc.), it is recommended that the “Create pH Measurement Anytime pH Falls Below Threshold” function be selected, as the data of the RE include many pH-only REs that do not alter the impedance value. Regarding the Exclude/Include Meal Periods function, it is recommended to select the “Exclude Meal Periods” for patients who are likely to consume acidic foods or beverages that may otherwise render the evaluation of GER inaccurate. In contrast, selection of the ‘Include Meal Periods’ is recommended for patients who are likely to consume non-acidic foods or beverages, to save time in the analysis of the MII-pH recorded data. Analyses performed during meals are excluded by including the initiation and completion times of the meal. When analyzing patients who receive enteral feeding with food not passing though the esophagus; that is, via a nasogastric tube or gastrostomy, it is alternative way to conduct MII-pH analysis without inputting the meal time.

In the pH classification function, it is recommended to select the “Acid/non-acid” for clinical purposes. A manual analysis without checking the “gastric pH analysis” can be performed easily in patients who do not require examination of gastric pH. Checking “Analyze Gas” is unnecessary for regular examinations because mixed RE (air and liquid), which is necessary to perform regular analyses, is detected automatically without checking, and several gas RE events when checking it in some patients forces the analyst to conduct a time-consuming analysis. However, evaluating gas reflux is important when the relationship between gas-related symptoms and GER needs to be assessed [7]. Therefore, the “Analyze Gas” function should be checked depending on the symptoms.

#### Manual analyses

Currently, there is no software program that can accurately analyze the MII-pH record of pediatric patients. Reports on pediatric MII-pH analyses have indicated that auto-analyses demonstrate a sensitivity and specificity of merely 94% and 74%, respectively, and auto-analyses tend only to detect an excessive number of REs [8]. In pediatric patients with many MII-pH REs that are difficult to analyze, manual analysis after auto-analyses is recommended to obtain accurate MII-pH results. A manual analysis can also facilitate recognition of the characteristics of the esophageal motility pattern. However, certain aspects of manual analyses can differ among analysts. These differences are particularly pronounced in cases with low baseline or rapidly changing esophageal impedance values caused by crying or air swallowing [9].

Manual analyses should be conducted to confirm the detection of RE by AutoScan™. When these REs are incorrectly detected, the marking as an RE should be deleted. However, when the AutoScan™ misses their detection altogether, the analyst must add the marking of REs in the waveform of MII-pH. It is important for the analyst to avoid an “over-diagnosis”. Patients who exhibit extra-esophageal symptoms must be evaluated for proximal REs. Therefore, the analyst should confirm whether an observed decrease in the impedance value reaches the channels placed in the proximal esophagus. In patients whose waveform is difficult to analyze, the analyst can accurately analyze the RE using the “color contour” mode.

The finding of numerous air movements in the waveforms indicates that conditions, such as gastric belching, supra-gastric belching, and air swallowing should be considered. Manual analysis of the impedance waveform pattern is useful for the diagnosis of such gas-related conditions.

In patients whose resting baseline impedance value in the lower esophagus is < 500 Ω, the analyst should evaluate the pH data only, as the evaluation of GER from the impedance records is inaccurate.

### Analysis report

The type of catheter used, circumstances under which the examination was performed, the analysis method and the results, and the interpretation of the analysis results based on the clinical symptoms should be included in the analysis report. If possible, the recommended treatment and additional examinations should also be added.

The parameters that should be included in the analysis report are as follows:

- pH
  - Reflux Index (%)
  - Number of pH RE
  - Number of pH RE > 5 min
  - Acid (chemical) clearance time (s)

- Impedance/pH
  - Number of REs (total, acid, non-acid)
  - Number of proximal REs (total, acid, non-acid)
  - Bolus exposure index (%)
  - Bolus clearance time (s)
  - Parameters of the relationship between symptoms and GER [4]

### Criteria for abnormal GER according to MII-pH

The criteria for abnormal GER according to MII-pH are as follows:

Abnormal GER: positive if any of the points below are observed:

1. Reflux Index ≥ 10% in patients aged < 1 year
  ≥ 5% in patients aged ≥ 1 year
2. Number of total liquid and mixed REs
  > 100 in patients aged < 1 year
  > 70 in patients aged ≥ 1 year

Significant relationship between GER and symptoms: positive if any of the points below are observed:

1. Symptom index (SI) ≥  50%.
2. Symptom association probability (SAP) ≥  95%.

## Discussion

Several studies have evaluated the normal data of MII-pH in healthy adults [10–12] and also the nearly normal data of MII-pH in neonates [13], infants, and children [14], albeit with relatively small sample sizes (Table 1). However, an analysis of MII-pH normal data in healthy children has not been performed. Currently, there is no international consensus regarding the criteria for abnormal GER based on MII-pH data in children. Of note, the German Pediatric Impedance Group (G-PIG) suggested new criteria for abnormal GER based on MII-pH data obtained from the analysis of 700 pediatric cases [15] and several studies have used these criteria. In 2016, Safe et al. reported another set of criteria, which included the SAP in addition to the SI. However, this parameter was not included in the parameters of the AutoScan™ when the G-PIG reported the criteria [16]. In Japan, there has been no update since the criteria for the cutoff value of the Reflux Index in children was first established by pH monitoring as a “Reflux Index > 4.0%” in 1998 [17]. Furthermore, several Japanese experts have recently indicated that the cutoff value of the “Reflux Index > 4.0%” seems much lower than values reported in Western countries (i.e., 5.0–7.0%) [18]. Moreover, the cutoff value of RI limited to infantile patients in Japan has not been established. Such discrepancies can become controversial among experts in Western countries and those in Japan. Therefore, following extensive discussion, the members of the Japanese-PIG reached a consensus that the cutoff value of RI in Japan should be adjusted to that recommended in Western countries (5.0%) to encourage the dissemination of studies on MII-pH from Japan to the global medical community. We recommended Japanese criteria for abnormal GER according to the measurement of MII-pH by consulting recently published international pediatric MII-pH reports. However, these criteria remain at a tentative status because they were not based on data obtained from studies involving Japanese children.

**Table 1:** Published normal values of multichannel intraluminal impedance-pH measurements (MII-pH) in neonates, infants, children, and adults

	References	Subjects (no.)	NoRE (total) (no.)	NoRE (acid) (no.)	NoRE (w-acid) (no.)	NoRE (alk) (no.)	MACT (s)	MBCT (s)	BEI (%)
Preterm neonate	López-Alonso et al.	21	71 (100.7)	n.r.	n.r.	n.r.	n.r.	n.r.	0.73 (1.21)
Child (1.3–17 years)	Mousa et al.	71	21 (71)	14 (55)	6 (34)^a^		n.r.	15 (32)	0.6 (2.4)
Infant (3 weeks–11.9 months)	Mousa et al.	46	54 (93)	20 (48)	32 (67)^a^		n.r.	13 (20)	1.4 (2.9)
Adult	Shay et al.	60	30 (73)	18 (59)	9 (26)	0 (1)	23	11	0.5 (1.4)
Adult	Zerbib et al.	68	44 (75)	22 (50)	11 (33)	3 (15)	34	11	n.r. (2)
Adult	Zentilin et al.	25	16 (48)	18 (51)	14 (38)	4 (18)	28	12	n.r.

*MII-pH* multichannel intraluminal impedance-pH measurements, Numbers are presented as medians (95th percentile), *NoRE (total)* number of total reflux episodes, *NoRE (acid)* number of acid reflux episodes, *NoRE (w-acid)* number of weakly acid reflux (defined as a reflux with a pH ≥ 4.0 but < 7.0) episodes, *NoRE (alk)* number of weakly alkaline reflux (defined as a reflux with a pH ≥ 7.0) episodes, *MACT* mean acid clearance time, *MBCT* mean bolus clearance time, *BEI* bolus exposure index, *n.r.* not reported

^a^Number of non-acid reflux episodes

In some institutions in Japan, a RI > 4.0% has recently been used as a criterion for the indication of the fundoplication procedure, particularly in neurologically impaired children. This tendency is observed despite the following statement in the guideline for pediatric 24-h esophageal pH monitoring: “it is vital that the diagnosis of GERD be made comprehensively by considering the simultaneity between the observed symptoms and the reflux events during pH monitoring, irrespective of RI > 4.0%”. We reported that the number of REs: 70, corresponds to GERD with RI of 9.2%, in which neurologically impaired patients suffer severe acid exposure [19]. Therefore, this MII-pH parameter may be more useful than RI in determining the indication for the fundoplication procedure.

pH-only REs, which are unique REs distinguishable by MII-pH, account for more than a quarter of the total number of pH REs in pediatric patients [20]. Of note, adult patients show a relatively small number of pH-only REs [20]. Although several possible mechanisms underlying pH-only RE have been suggested, such as short-column acid reflux [21], low volume [22], residuals of previous impedance detectable acid RE [23], esophageal shortening [24], and artifacts from swallowing acidic contents, this type of RE has been shown to contribute significantly to total esophageal acid exposure [25]. Therefore, when there is a large gap in the total number of pH REs and REs detected by impedance, incorporating the evaluation of pH-only REs may be useful for clarifying the cause of symptoms in pediatric patients, which cannot be identified using only automatically calculated MII-pH parameters.

Although MII-pH measurement has been adopted widely in Japan and overseas, the methodology of this analysis and the interpretation of its results are not well-established in Japan versus Europe and the United States. Furthermore, discrepancies and lack of reproducibility have been noted in the results of MII-pH analyses among examiners. Ideally, this examination should be performed by a well-trained and experienced analyst. Recently, in clinical research utilizing MII-pH measurement, interesting findings have been reported by Japanese pediatric surgeons [26–28]; for example, the utility of MII-pH for evaluation of the fundoplication effect [27] and the baseline impedance value known as potent parameters for estimating esophageal mucosal inflammation [28]. Improvements in the accuracy and standardization of analysis among examiners and institutions may be necessary to expand the application of MII-pH measurement in Japan. This may be achieved by conducting regular educational seminars on MII-pH measurement in this setting.
